# Post-herniotomy Ischemic Orchitis in a Child: A Silent Threat

**DOI:** 10.7759/cureus.97813

**Published:** 2025-11-25

**Authors:** Muhammad Mudasir Saleem, Mishal Pervaiz, Hira Shamim, Maida Muazzam, Mir M Rai, Ajwa Kaleem, Rehmat Kaleem, Maidah Javaid

**Affiliations:** 1 Pediatric and General Surgery, Combined Military Hospital Lahore, Lahore, PAK; 2 Anaesthesiology, Punjab Rangers Teaching Hospital, Lahore, PAK; 3 Pediatric Surgery, Combined Military Hospital (CMH) Lahore Medical College and Institute of Dentistry, Lahore, PAK; 4 Pediatric Surgery, Shifa Tameer-E-Millat University Shifa College of Medicine, Islamabad, PAK; 5 Surgery, Lahore General Hospital, Lahore, PAK; 6 Pediatric Surgery, Mayo Hospital, Lahore, PAK; 7 Community Medicine, Combined Military Hospital (CMH) Lahore Medical College and Institute of Dentistry, Lahore, PAK

**Keywords:** doppler ultrasonography, inguinal hernia repair, ischemic orchitis, orchiectomy, pediatric herniotomy, postoperative complication, testicular ischemia

## Abstract

Ischemic orchitis is an uncommon but potentially serious complication of pediatric inguinal hernia repair. Although most postoperative alterations in testicular blood flow are transient, persistent ischemia may progress to infarction and testicular loss. Doppler ultrasonography is the key diagnostic tool for early recognition. Awareness of this rare complication, careful surgical technique, and prompt evaluation of postoperative scrotal symptoms are essential to optimize outcomes and preserve testicular viability in children undergoing herniotomy. We report a six-year-old boy who developed acute scrotal pain and swelling following elective right herniotomy. Doppler ultrasound revealed markedly reduced intratesticular blood flow consistent with ischemic orchitis. Despite initial conservative management, progressive ischemia necessitated scrotal exploration, which revealed a non-viable testis requiring orchiectomy.

## Introduction

Inguinal herniotomy is one of the most frequently performed pediatric surgical procedures and is generally associated with minimal morbidity [1]. However, rare but serious complications such as ischemic orchitis can compromise testicular viability and, in extreme cases, can result in orchiectomy [2]. Transient postoperative alterations in testicular perfusion have been documented, particularly in the early period after surgery, with recovery usually occurring within months [3]. Advances in Doppler and microvascular imaging have enabled better detection of subtle vascular disturbances following hernia repair in children [4]. While most changes are reversible, sustained vascular compromise may lead to testicular atrophy or ischemia [5]. Reports of complete testicular infarction after herniotomy in children are exceedingly rare, making such cases valuable for raising awareness. This underscores the importance of careful surgical technique, vigilant postoperative assessment, and early Doppler evaluation when acute scrotal symptoms occur [6].

## Case presentation

A six-year-old boy underwent an elective open right inguinal herniotomy as a day-care procedure at another healthcare facility. According to the mother, scrotal swelling appeared approximately 18 hours after surgery and progressively increased. The operating surgeon was contacted and advised oral analgesics and bed rest; however, due to worsening symptoms, the parents presented to our clinic approximately 24 hours after the first procedure. The operative notes, obtained from the primary surgeon, described standard hernial sac dissection with high ligation and no reported intraoperative complications, with no mention of difficult dissection, excessive cord traction, or vascular compromise.

**Figure 1:**
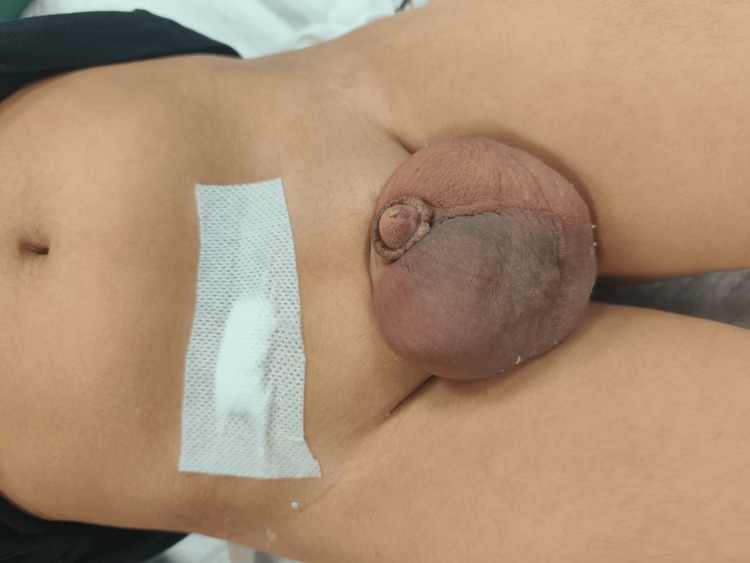
Scrotal swelling limited to right hemiscrotum on presentation. Groin dressing over previous operative site is also evident.

On examination, the child had a tender right scrotal swelling measuring 6 × 8 cm involving the entire testis, with a smooth, firm consistency. The epididymis was not separately palpable, and the overlying scrotal skin showed erythema (Figure 1). The child was offered open inguinal exploration at our center, but the parents opted to wait due to domestic reasons.

**Figure 2:**
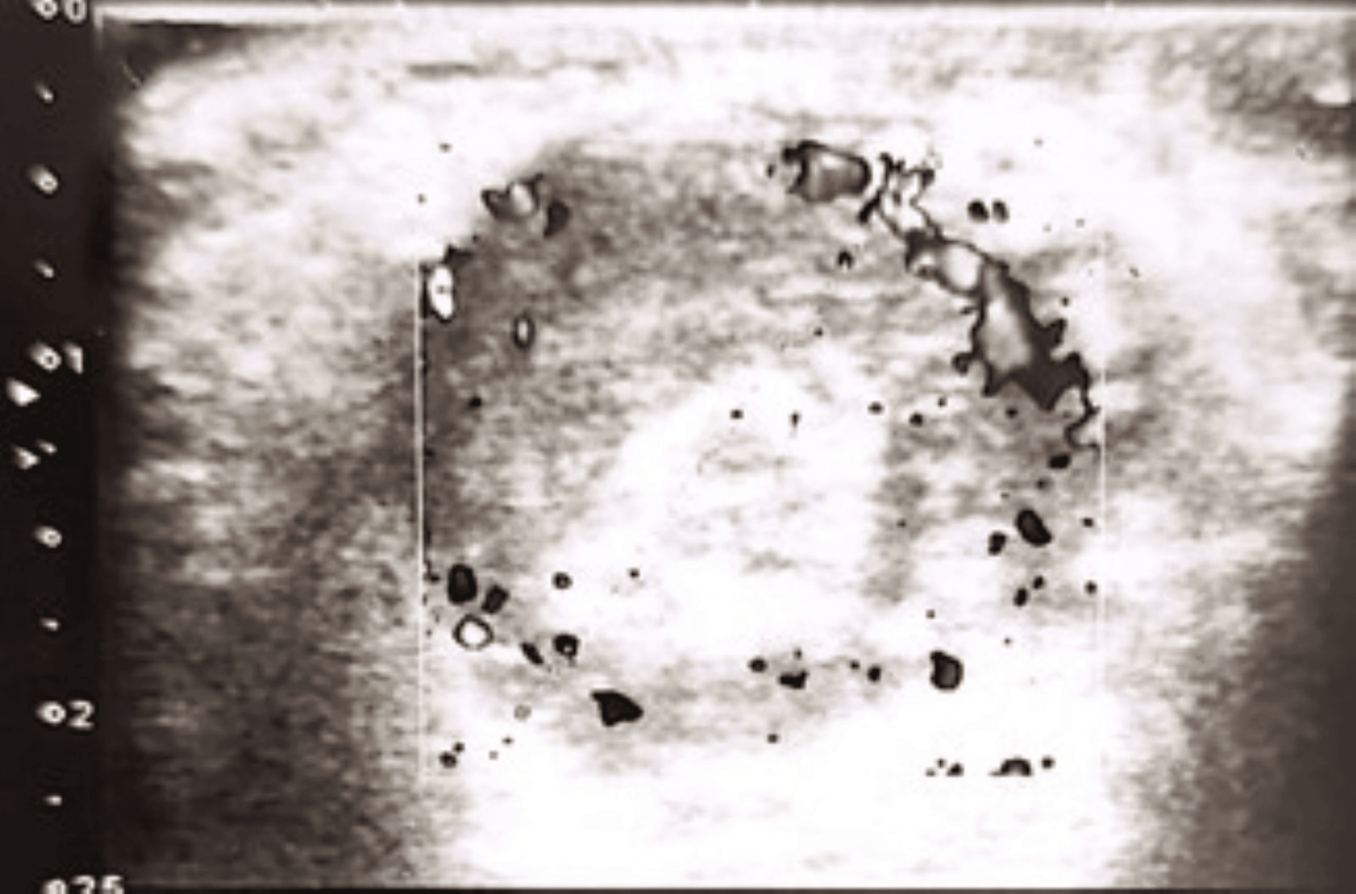
Doppler ultrasound showing reduced central blood flow with preservation of peripheral blood flow in right testis.

An urgent Doppler ultrasound was obtained, which showed an enlarged right testis with heterogeneous echotexture and markedly reduced intratesticular blood flow compared with the contralateral side, with preservation of peripheral blood flow and thickening of the overlying scrotal skin, confirming the diagnosis of ischemic orchitis. There was no torsion of the testis or spermatic cord (Figure 2).

**Figure 3:**
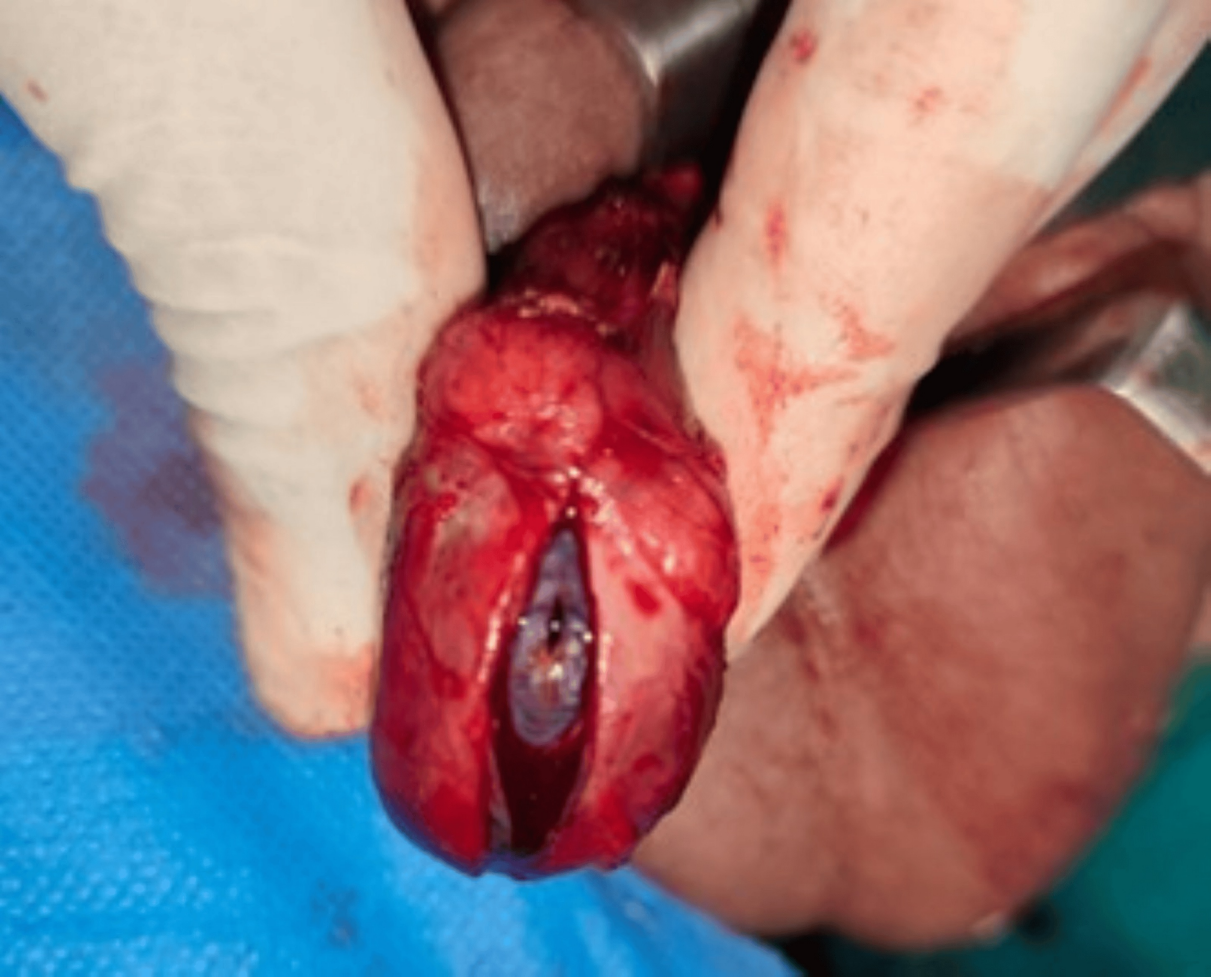
Operative findings showing non-viable right testis.

The parents were counseled in detail regarding the diagnosis of ischemic orchitis and its potential complications. The child was admitted and started on intravenous broad-spectrum antibiotics, analgesics, bed rest, and scrotal elevation. Given that the initial Doppler showed preserved cord flow and no evidence of torsion, a period of close observation was undertaken. Serial clinical monitoring over the next 12 hours demonstrated progressive scrotal enlargement, increasing pain, and intermittent fever. A follow-up Doppler ultrasound was performed approximately 12 hours after admission and showed further enlargement of the right testis, with a significant reduction in intratesticular blood flow compared with baseline. In view of these worsening findings and concern for evolving infarction, urgent scrotal exploration was undertaken under general anesthesia. A standard midline raphe incision was used, providing adequate exposure even in the pediatric scrotum. Intraoperatively, the scrotal skin was markedly edematous, the spermatic cord was congested and swollen, and the right testis was enlarged, firm, and showed patchy blackish discoloration on opening the tunica albuginea (Figure 3).

**Figure 4:**
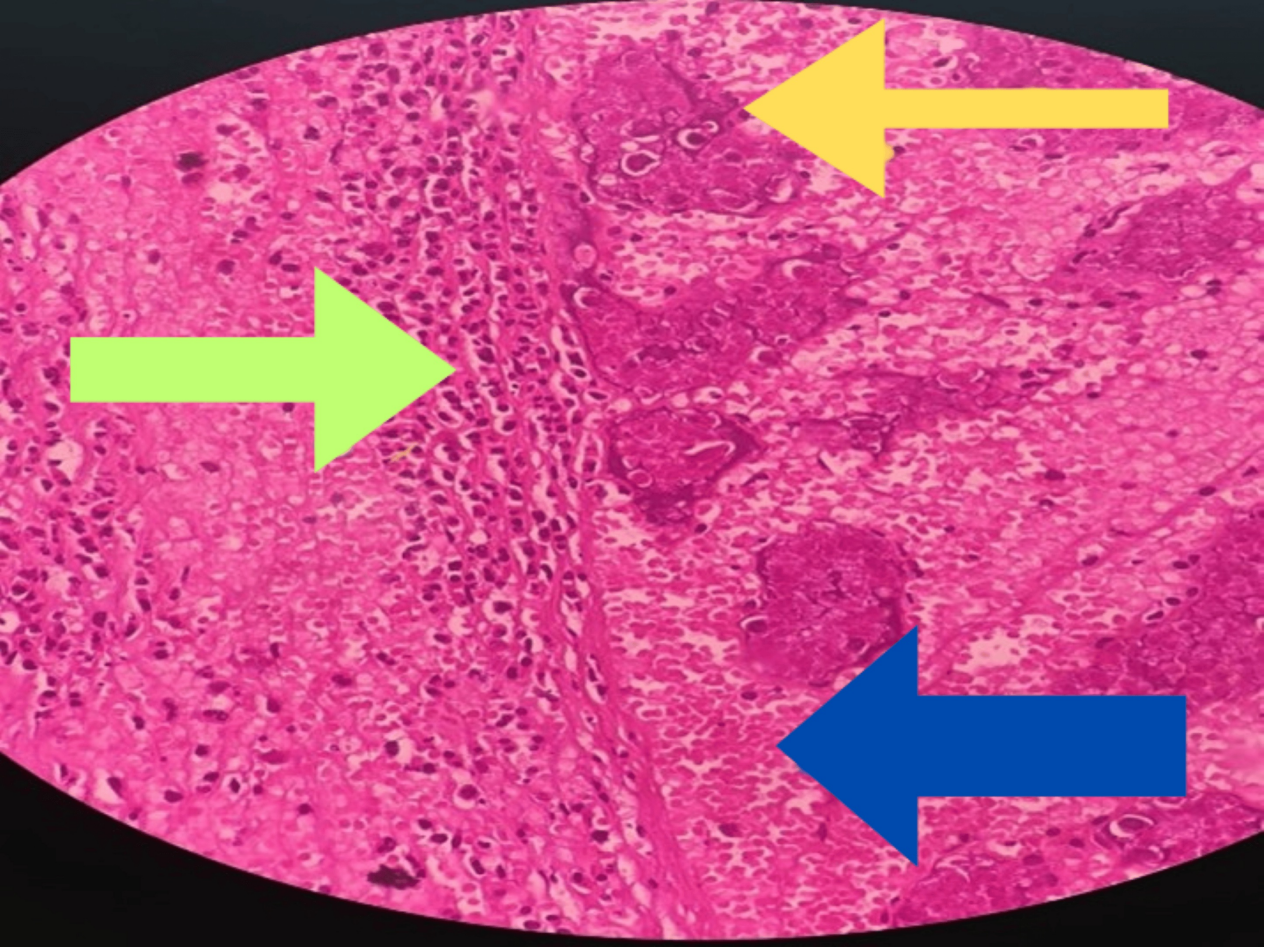
Specimen histopathology report consistent with testicular infarction. Yellow arrow: atrophic seminiferous tubules. Green arrow: inflammatory cells. Blue arrow: hemorrhage.

A right orchidectomy was performed after obtaining informed consent from the parents. The testicular vessels were ligated at a high level to ensure complete removal of the non-viable testis. Exploration of the contralateral testis revealed a mild hydrocele on opening the tunica vaginalis. A three-point orchidopexy was performed on the contralateral testis using non-absorbable sutures to prevent future torsion. A small closed-suction drain (14 Fr) was placed to prevent postoperative fluid collection, and the wound was closed in layers. Histopathology of the excised testis revealed atrophic seminiferous tubules with hemorrhage, congestion, and a mixed inflammatory infiltrate of neutrophils and lymphocytes, consistent with testicular infarction (Figure 4).

The child showed uneventful recovery and was discharged on the third postoperative day after removal of the drain. Follow-up at two weeks, one month, and three months showed a completely healed scrotal wound with a normal contralateral testis.

## Discussion

This case of a six-year-old child with acute post-herniotomy ischemic orchitis culminating in orchidectomy highlights several important clinical lessons. While transient reductions in testicular perfusion are commonly reported after pediatric hernia repair, complete infarction is rare, particularly in patients presenting early. In our case, despite timely presentation and initiation of conservative measures, testicular perfusion deteriorated rapidly, necessitating surgical exploration. Post-herniotomy ischemic orchitis is multifactorial.

Key contributors include injury to the testicular artery or pampiniform plexus, excessive traction on the spermatic cord, extensive cord skeletonization during sac dissection, postoperative hematoma, and inadvertent vascular ligation [7]. In the index case, operative notes from the primary surgeon confirmed a standard open high ligation, classic herniotomy, without reported intraoperative complications or excessive cord traction. No hematoma was documented at the initial surgery, but the possibility of microvascular injury leading to rapid ischemia cannot be excluded.

Pareja et al. [8] described a similar post-hernia ischemic event managed conservatively with preserved testicular viability, in which Doppler showed reduced but not absent flow. In contrast, our patient exhibited near-complete loss of Doppler signal, illustrating that conservative management is unlikely to succeed in the presence of severe vascular compromise. Dellabianca et al. [9] described chronic testicular ischemia in adults with diffuse hypoechogenicity and absent intratesticular Doppler signal; our patient’s ultrasound findings were similar, though cremasteric vessel hypertrophy was absent, consistent with the acute nature of ischemia. While Dellabianca et al. described chronic testicular ischemia in adults without echogenicity changes on ultrasound, our pediatric case represents an acute ischemic event with rapid development of intratesticular echogenic changes and complete loss of Doppler flow, highlighting that acute ischemia can present differently and progress more rapidly in children.

Nakamura [10] reported post-laparoscopic herniotomy hematocele causing ischemia requiring orchidectomy due to delayed presentation; our case differs in that presentation was early, yet the initial ischemic insult was already severe, emphasizing that rapid progression can occur even with timely evaluation.

Several preventative measures can minimize the risk of ischemic orchitis. These include using a classic high ligation carefully while avoiding unnecessary dissection around the cord, minimizing traction on the spermatic cord and avoiding skeletonization of cord vessels unless required for complete sac excision, and taking care to preserve the testicular artery and pampiniform plexus to prevent inadvertent ligation or cautery near the vessels. Meticulous hemostasis is essential to prevent postoperative hematoma or cord compartment syndrome. Early Doppler evaluation in children who develop postoperative pain, swelling, or erythema can help detect perfusion compromise before irreversible injury occurs.

This case underscores that even early presentation does not guarantee testicular salvage if the initial ischemic insult is severe. Doppler evidence of complete arterial flow loss is a poor prognostic marker, highlighting that some cases of ischemic orchitis will inevitably require orchidectomy despite prompt intervention.

## Conclusions

This case highlights ischemic orchitis as a rare but serious postoperative complication of pediatric inguinal herniotomy, which in this patient culminated in testicular infarction and orchidectomy. Importantly, it demonstrates that even early recognition and conservative management may not be sufficient to salvage the testis when vascular compromise is complete.

The outcome in this patient was compounded by delayed intervention and underscores that postoperative scrotal complications should be evaluated promptly by a trained pediatric surgeon. Careful surgical technique to avoid vascular injury, early clinical assessment of postoperative scrotal symptoms, and timely use of Doppler ultrasonography are essential to detect and manage vascular compromise. While conservative management may succeed when partial perfusion persists, surgical exploration should not be delayed in cases of complete or progressive ischemia. Vigilance, specialized surgical care, and awareness of the potential severity of ischemic orchitis are crucial to prevent testicular loss and preserve future fertility.
